# Application of electrospun piezoelectric nanofibers in wound healing

**DOI:** 10.1093/burnst/tkag032

**Published:** 2026-04-30

**Authors:** Jing Wang, Jianhua Feng, Chengzhi Zhang, Xiankui Gao, Changjun Jia, Tianming Zhao, Seeram Ramakrishna, Hongye Guan

**Affiliations:** Zhejiang Provincial Key Laboratory of Pancreatic Disease, The First Affiliated Hospital, Zhejiang University School of Medicine, Hangzhou 310003, Zhejiang, People’s Republic China; The First Affiliated Hospital, Heilongjiang University of Chinese Medicine, No. 26 Heping Road, Xiangfang District, Harbin 150040, Heilongjiang, People's Republic of China; Zhejiang Provincial Key Laboratory of Pancreatic Disease, The First Affiliated Hospital, Zhejiang University School of Medicine, Hangzhou 310003, Zhejiang, People’s Republic China; State Key Laboratory of Robotics and Intelligent Systems, Shenyang Institute of Automation, Chinese Academy of Sciences, Shenyang, 110016, Liaoning, People’s Republic China; Zhejiang Provincial Key Laboratory of Pancreatic Disease, The First Affiliated Hospital, Zhejiang University School of Medicine, Hangzhou 310003, Zhejiang, People’s Republic China; General Education Department, Qilu Medical University, Zibo 255300, Shandong, People’s Republic China; State Key Laboratory of Robotics and Intelligent Systems, Shenyang Institute of Automation, Chinese Academy of Sciences, Shenyang, 110016, Liaoning, People’s Republic China; College of Design and Engineering, National University of Singapore, Singapore 117575, Singapore; Zhejiang Provincial Key Laboratory of Pancreatic Disease, The First Affiliated Hospital, Zhejiang University School of Medicine, Hangzhou 310003, Zhejiang, People’s Republic China

**Keywords:** Biomaterial, Piezoelectric nanofibers, Electrospinning, Electrical stimulation, Wound healing

## Abstract

Wound healing is essential for maintaining organismal homeostasis and barrier function, posing significant challenges particularly in the management of hard-to-heal wounds such as diabetic ulcers. This review systematically summarizes the functional properties of piezoelectric electrospun biomaterials and their biological mechanisms in promoting wound healing via electrical stimulation (ES). Piezoelectric nanofiber wound dressings fabricated by electrospinning technology offer dual advantages: on one hand, their unique fibrous architecture enables adaptation to irregular wound contours, facilitates rapid hemostasis, and provides an effective barrier against microbial invasion; on the other hand, their inherent piezoelectric effect converts micromechanical deformations into localized electrical signals in real time, thereby modulating cellular behaviors and accelerating tissue repair. This review highlights recent studies elucidating the mechanisms by which ES promotes wound healing, and the functional characteristics of piezoelectric fibrous dressings. It aims to establish a theoretical foundation for the rational design and performance optimization of next-generation intelligent piezoelectric wound dressings, thereby advancing their clinical translation and industrial development.

## Highlights

- Electrical stimulation therapy contributes distinctively and positively to all four stages of wound healing.
- The diverse species, dimensions, and sizes of piezoelectric electrospun biomaterials enable their tunable physical and electrical properties.
- Piezoelectric electrospun biomaterials serve as key functional materials in diverse electrotherapy systems for wound healing.
- Piezoelectric electrospun biomaterials contribute new insights to the field of wound healing promotion and bring to light current challenges.

## Background

Skin trauma, a common pathological condition caused by physical, chemical, or biological factors, leads to tissue damage and functional impairment. It encompasses various types, such as skin injuries, burns, and ulcers, with clinical manifestations including local pain, bleeding, and swelling. Severe cases can result in infection, sepsis, or even organ dysfunction [1–3]. The growing aging population and rising prevalence of chronic diseases like diabetes are making hard-to-heal wounds a significant global public health challenge [4, 5]. Skin trauma causes both physical suffering and a diminished quality of life from complications like infection and scarring [2, 6]. The complex process of wound healing occurs via four consecutive stages: hemostasis, inflammation, proliferation, and remodeling [7–9]. However, conventional treatments mainly rely on physical coverage and adjunctive drugs, and are unable to actively regulate the healing process. They struggle to effectively promote tissue regeneration, showing limited efficacy, especially in chronic and complex wounds [9, 10]. Therefore, the development of efficient and safe methods for trauma repair to achieve rapid wound healing is crucial for improving patient outcomes.

Electrical stimulation (ES) promotes wound healing by activating calcium ion channels and the extracellular signal-regulated kinase (ERK) pathway. This activation enhances fibroblast proliferation, migration, and differentiation, which in turn accelerates tissue regeneration [11, 12]. However, the safe and controllable application of ES to the wound site remains a challenge. ES therapy can be realized through various technologies, including piezoelectricity, triboelectricity, photoelectricity, wireless transmission, and batteries. Among these, piezoelectric technology is a crucial method, as it directly converts minute mechanical energy from the environment or the body into electrical energy, thereby generating ES to surrounding cells and tissues. To effectively implement this technology, electrospun biomaterials serve as an ideal platform. These functional materials, fabricated via electrospinning, possess micro/nano-fiber structures specifically designed for biomedical applications such as tissue engineering and wound repair. Their core advantages lie in their biomimetic physical structure and highly customizable chemical and biological functions. Building on these properties, piezoelectric nanofibers have been developed as advanced functional materials [13]. Typically prepared from intrinsic piezoelectric materials or composites via electrospinning, they combine piezoelectric functionality with the mechanical flexibility, high specific surface area, and biomimetic structural characteristics of nanofibers, making them ideal materials for constructing flexible self-powered sensors, nanogenerators, and smart biomedical devices. Fabricated via a high-voltage electric field, these fibers leverage their direct piezoelectric effect, converting mechanical signals into electrical signals, to enable continuous ES [14, 15]. Piezoelectric performance originates from the material's intrinsic non-centrosymmetric crystal structure or molecular arrangement. As a unique physical form, nanofibers, through their size and structural effects, can significantly enhance the actual performance of piezoelectric materials, making them more suitable for wound healing scenarios. These electrical signals are highly similar to the physiological electrical signals naturally generated by the human body, allowing them to precisely mimic the endogenous ES environment during wound healing and effectively guide cell migration, proliferation, and tissue regeneration [16, 17]. Piezoelectric nanofiber-based dressings leverage a structure that mimics the native extracellular matrix (ECM), thereby promoting effective cell adhesion. Moreover, they promote cell proliferation and tissue repair through endogenous ES. This overcomes the limitations of traditional methods in incorporating this therapeutic modality and opens new avenues for understanding healing mechanisms and developing active regulation strategies [18].

**Figure 1:**
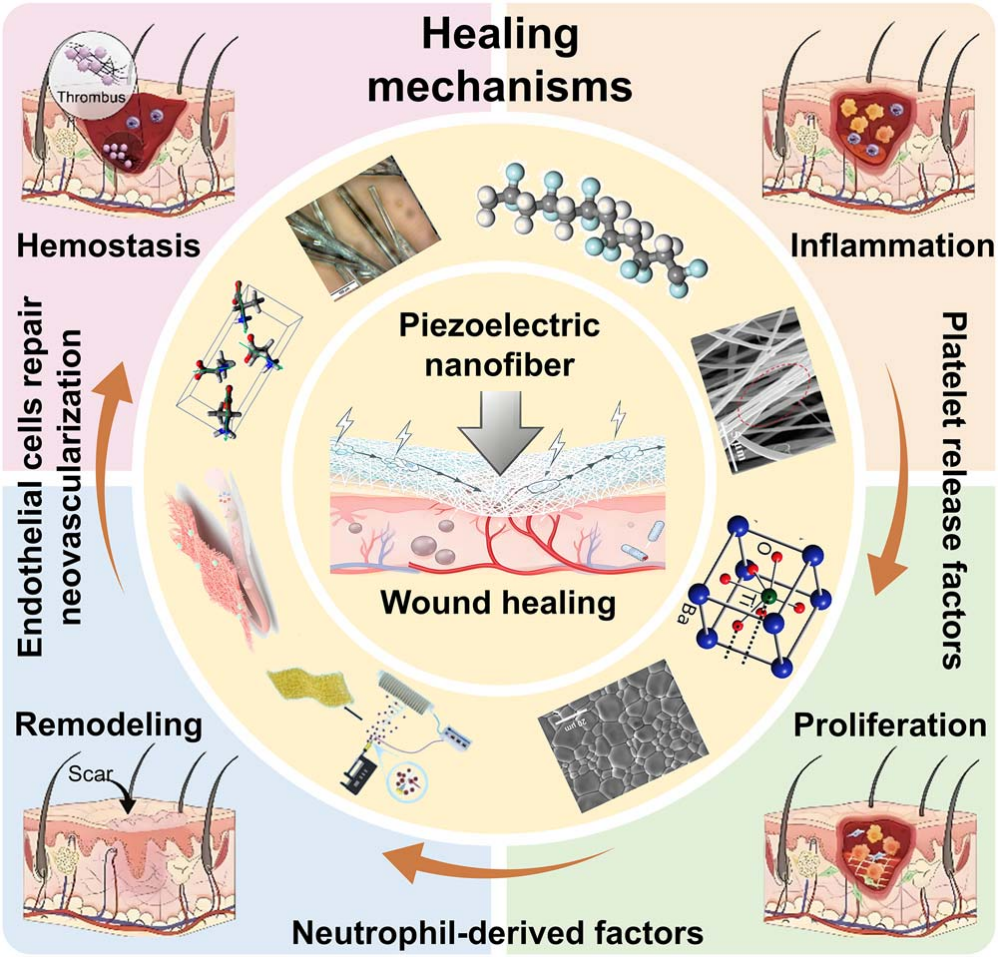
Applications of electrospun piezoelectric nanofibers in wound healing: from healing mechanisms and piezoelectric biomaterials to diverse applications. Natural polymers: reproduced with permission [19]. Copyright 2019, American Physical Society. Reproduced with permission [20]. Copyright 2018, Springer Nature. Synthetic polymers: reproduced with permission [21]. Copyright 2022, John Wiley and Sons. Reproduced with permission [22]. Copyright 2020, Springer Nature. Inorganic materials: reproduced with permission [23]. Copyright 2025, Springer Nature. Reproduced with permission [24]. Copyright 2023, John Wiley and Sons. Complex materials: reproduced with permission [25]. Copyright 2025, Elsevier. Reproduced with permission [26]. Copyright 2025, Elsevier. Physiological stages**:** reproduced with permission [27]. Copyright 2024, Elsevier

We systematically summarize the research progress and application prospects of piezoelectric nanofibers for wound healing. This review begins with the physiology of skin wound healing and then examines the mechanistic role of ES in facilitating repair. Building on this, it systematically examines the characteristics and piezoelectric effects of typical piezoelectric biomaterials, including natural polymers, synthetic polymers, inorganic materials, and composite materials. Subsequently, it details the innovative application forms of piezoelectric nanofibers in wound dressings and summarizes their specific therapeutic efficacy in skin trauma repair. Finally, this review outlines future research directions for the field. These include elucidating the pro-healing mechanisms of ES, optimizing dressing performance and comfort, promoting clinical translation, and exploring synergistic therapies and analgesic effects. Overcoming these barriers is an essential step toward the industrialization and clinical adoption of piezoelectric nanofibers. This review is intended to provide a theoretical reference and practical guidance for the development and application of high-performance piezoelectric nanofibers (Fig. 1) [19–27].

## Review

### Physiological stages of skin wound healing

**Figure 2:**
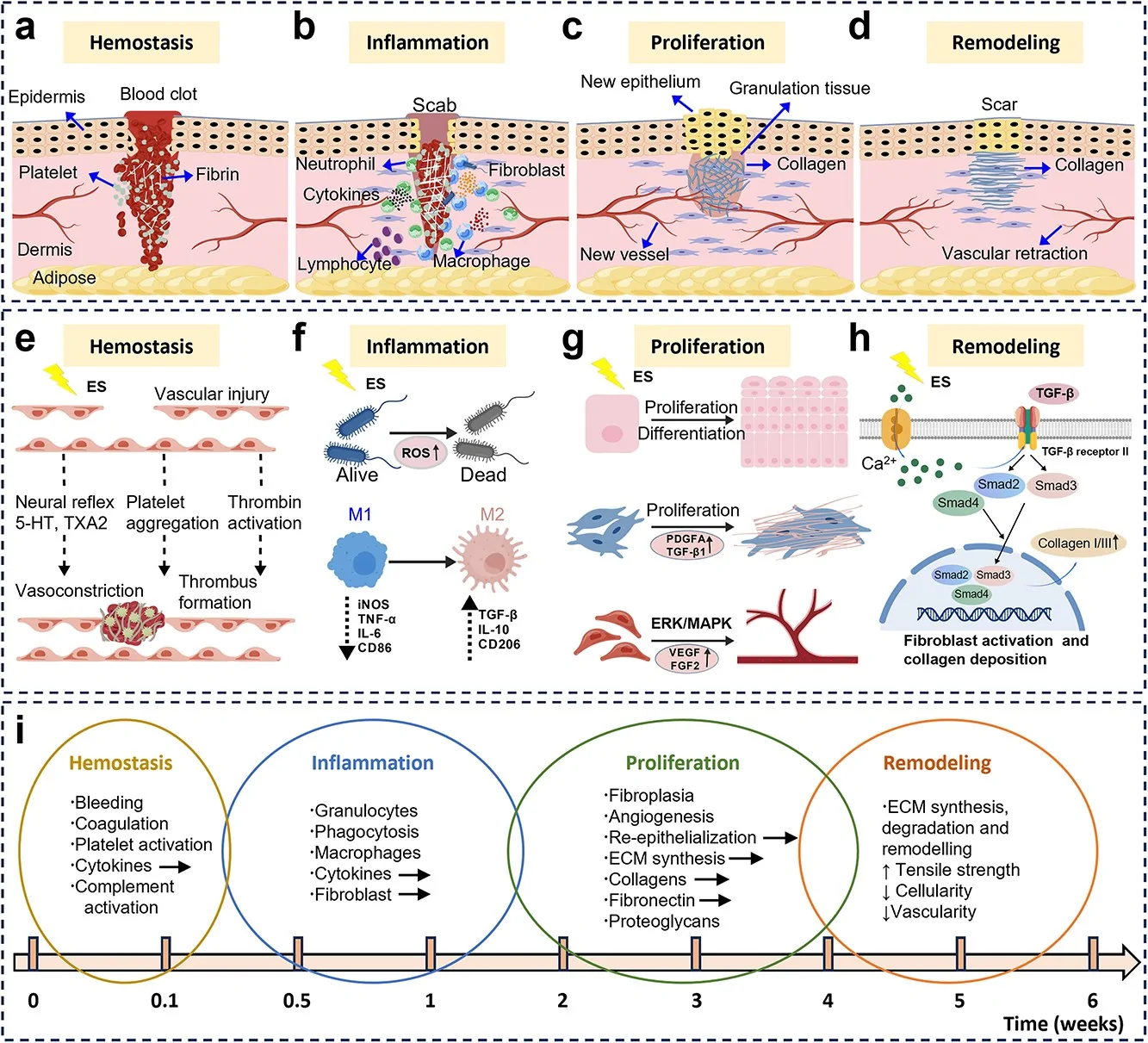
Schematic illustration of physiological wound healing process and electrical stimulation regulatory mechanisms. (**a**–**d**) schematic diagrams of four stages of skin healing: (a) Hemostasis phase, (b) inflammatory phase, (c) proliferative phase, and (d) remodeling phase. (**e**) Hemostasis phase: electrical stimulation promotes neural reflexes, platelet aggregation, and thrombin activation to form platelet thrombus. (**f**) Inflammatory phase: electrical stimulation induces ROS elevation and macrophage M2 polarization to exert antibacterial effects. (**g**) Proliferative phase: electrical stimulation accelerates re-epithelialization, fibroblast proliferation and collagen deposition, and promotes angiogenesis. (**h**) Remodeling phase: electrical stimulation enhances fibroblast activity through the TGF-β/Smad2/3/4 axis to accelerate collagen remodeling. (**i**) Timeline diagram of the four stages of wound healing. Created with BioGDP.com

The skin is the most expansive organ in the human body, consisting of the epidermis, dermis, and subcutaneous tissue. Its primary functions include acting as a barrier to resist physical and chemical damage, facilitating metabolism, excreting waste, regulating body temperature and immunity, and maintaining bodily homeostasis [28]. Skin wound healing is a complex and orderly physiological process that proceeds through four overlapping yet distinct stages: hemostasis, inflammation, proliferation and remodeling (Fig. 2a–d and i) [9]. During this process, multiple growth factors and cytokines work together to promote immune cell differentiation, clear cellular debris, defend against infection, stimulate angiogenesis and cell proliferation, propelling the wound-healing process forward [29, 30]. Acute wounds refer to those that follow the normal healing timeline and proceed orderly through four stages, typically healing within 4 weeks; chronic wounds are defined as those that fail to restore anatomical and functional integrity after 3 months. Although both undergo hemostasis, inflammation, proliferation, and remodeling phases, chronic wounds are hindered by prolonged inflammation, reduced cellular activity, and matrix metalloproteinase imbalance [31]. Chronic wounds currently face challenges including persistent inflammation, impaired growth factor signaling, bacterial infection, and cellular senescence [32, 33]. This review focuses on piezoelectric materials that promote cell proliferation, migration, and angiogenesis through ES-effects that are therapeutically relevant to the proliferative phase of both acute and chronic wounds. Therefore, the cited literature encompasses evidence for ES efficacy in both wound types (Table 1) [34–52].

Studies have shown that piezoelectric therapy addresses these challenges by reactivating quiescent cells, upregulating endogenous growth factors, improving microcirculation, and generating reactive oxygen species (ROS) for antimicrobial effects [53, 54]. The molecular mechanisms involve mitogen-activated protein kinase (MAPK)/ERK, phosphatidylinositol 3-kinase (PI3K)/Akt, Wnt/β-catenin, and transforming growth factor-β (TGF-β)/Smad pathways, as well as mechanosensitive ion channel-mediated Ca^2+^ signaling, which coordinately promote cell proliferation, survival, migration, and collagen synthesis to facilitate chronic wound healing [17, 55–57].

#### Hemostasis phase

Hemostasis is the first stage of skin wound healing, occurring within seconds to minutes of injury (Fig. 2a). Skin damage stimulates local nerve reflexes, releasing vasoactive substances that cause vascular smooth muscle contraction, rapidly reducing blood flow [58]. Simultaneously, collagen beneath the endothelium is exposed. Platelets adhere to the collagen via von Willebrand factor and interact with glycoprotein IIb/IIIa receptors, becoming activated and forming an initial thrombus [59, 60]. Activated platelets release granule contents, providing phospholipid surfaces and procoagulant factors that accelerate the ignition of the intrinsic and extrinsic clotting pathways [61, 62]. Fibrin mesh reinforces platelet plugs to form stable thrombi, sealing vascular ruptures. Platelets are pivotal in the hemostatic phase and throughout the entire healing process, not only halting bleeding but also secreting diverse cytokines and chemokines [63, 64]. These recruit neutrophils and macrophages to the wound, initiating the next stage of wound healing [65].

#### Inflammatory phase

The inflammatory phase is the second stage of skin wound healing, occurring within hours to 2–5 days of injury (Fig. 2b). It is marked by the immune cells such as neutrophils, lymphocytes and macrophages pouring into the wound. Neutrophils spearhead the immune response, accumulating in large numbers within the initial 24 h before releasing apoptotic products and being replaced by macrophages [66]. Macrophages and other phagocytic cells appear in the wound to clear debris and bacteria. They infiltrate ~48 h after the wound occurs and continue until the end of the inflammatory phase [67]. Neutrophils and macrophages release multiple cytokines including interleukin-1α (IL-1α), tumor necrosis factor-α (TNF-α), TGF-β, and interleukin-6 (IL-6) collectively regulating inflammatory responses, cell chemotaxis, and tissue repair [68, 69]. Inflammation is a necessary stage of healing, but it may be prolonged due to bacterial infection, repeated trauma, and foreign bodies [70]. The duration of the inflammatory phase depends on the pathogen load within the wound microenvironment and the extent of necrotic tissue residue. If inflammation is disrupted or prolonged, it may lead to tissue damage, delayed proliferation, and the formation of chronic wounds and scars. When the local microbial load decreases and necrotic debris is fully degraded, the inflammatory signaling cascade weakens, and the wound enters the proliferative phase [71].

#### Proliferation phase

The proliferative phase is the third stage of skin wound healing, commencing 2–3 days after injury and lasting 2–3 weeks (Fig. 2c). This phase encompasses complex processes including epithelialization, angiogenesis, granulation and collagen deposition to fill the defect left after wound debridement. Re-epithelialization is characterized by the proliferation and infiltration of keratinocytes at the wound margin. Stem cells within hair follicles and apocrine glands differentiate into keratinocytes, digest the ECM through proteases, refill the basal layer, and migrate across the wound edges [72]. Angiogenesis is triggered at the moment of thrombus formation, with platelets releasing cytokines TGF-β, fibroblast growth factor (FGF), and vascular endothelial growth factor (VEGF), inducing endothelial cells to repair damaged blood vessels and initiating angiogenesis [73]. Angiogenesis takes place through a coordinated series of sprouting, looping, and pruning guided by cytokine gradient signals [74]. Angiogenesis is influenced by primary and secondary wound healing. In primary healing, wound edges are neat and tissue defects are minimal, allowing rapid angiogenesis. Secondary healing achieves vascularization through hypoxia, elevated lactate levels, and growth factor-induced granulation tissue formation. Furthermore, in secondary healing wounds, the area of epithelial loss is larger, and the wound is filled with ‘temporary granulation tissue’ composed of new blood vessels, immature collagen III, and fibroblasts [75]. Fibroblasts differentiate into myofibroblasts at this stage, contracting to pull the wound edges together [9, 76].

#### Remodeling phase

The remodeling phase is the fourth stage of skin wound healing, commencing in the first week post-injury and lasting for months to years (Fig. 2d). The core task of the remodeling phase is to transform the ‘temporary granulation tissue’ into a ‘permanent scar’, restoring tension and reconstructing the barrier. This phase begins with the replacement of temporary ECM and collagen III by collagen I, alongside the apoptosis of residual cells from the preceding stage [9]. As collagen I deposits accumulate, the wound's tensile strength increases, granulation tissue atrophies, and redundant blood vessels retract. A successful remodeling phase requires a dynamic equilibrium between synthesis and degradation, with the synthesis process being highly energy-dependent. As the scar gradually matures, vascularization declines and its color shift to gray. The remodeling phase is the longest, ultimately forming normal epithelium and promoting scar tissue maturation. Excessive fibrosis during this stage can give rise to hypertrophic scars or keloids [77].

### The potential mechanisms of electrical stimulation in wound healing

As an effective physical therapy, ES promotes wound healing at every stage of the process. The skin's intrinsic electrical potential also modulates wound healing. In normal skin, a trans-epidermal potential of 10–60 mV is maintained across the epidermis and subepidermal layers due to ion channel transport and cellular depolarization and repolarization [16]. When the skin is damaged, this potential is disrupted, creating an endogenous electric field directed toward the center of the wound. A voltage of 100–150 mV/mm develops between the wound and the adjacent intact skin, generating a wound current [78–81]. These endogenous electric fields play a crucial role in wound healing [82–84]. In their absence, the average wound healing rate decreases by ~25%, which has also driven the exploration of applications for ES in promoting wound healing [85, 86]. Since the end of the 20th century, ES therapy has been developed for wound treatment, particularly chronic wounds [87, 88]. Exogenous ES accelerates wound healing by applying direct current, alternating current, or pulsed currents through electrodes placed on or around the wound site, thereby mimicking or amplifying the wound's electrical potential [89, 90]. Research indicates that ES could increase perfusion, reduce infections, boost immunity, and accelerate skin wound healing [91]. Research indicates that ES promotes healing at various stages of acute and chronic wounds through different pathways, accelerating the healing process (Table 1). Moreover, Rabbani *et al.* conducted a systematic review analyzing the parameters of ES for promoting wound healing, indicating that the voltage output range of piezoelectric dressings is between 35 mV and 8 V, with current output ranging from 40 nA to 250 nA. They further emphasized that an electric field strength of 100–400 mV/mm can effectively promote cell migration and proliferation [92].

**Table 1:** Mechanisms by electrical stimulation promotes wound healing

Working mode	Signal type	Signal parameters	Stimulation duration	Stimulation days	Biological effects	Ref.
Constant voltage	Bipolar pulse	20 V/12.5 mA, 10 Hz, 1 ms, 1%	5 min	1	Vasoconstriction	[34]
	Bipolar pulse	150 V/120 mA, 10 Hz, 10 μs, 0.01%	5 min	1	Vasoconstriction	
	Single-phase pulse	3.72 kV/315 mA, 1 Hz, 600 ns, 0.00006%	5 s	1	Activate platelets	[35]
	Direct current	9 V/20–30 mA, Continuous, 100%	10s	1	Promote fibrin formation	[36]
	Single-phase pulse	~4000 V/cm, 5 μs	5 μs	1	Promote coagulation	[37]
	Bipolar pulse	~3000 V/cm, 1 Hz, 150 ns	76 μs	1	Promote coagulation	
	Pulsed direct current	200 mV, 10 Hz, 1 ms, 0.01%	30 min	10	Anti-inflammatory and Proliferation-promoting	[39]
	Alternating current	0.75 V	5 min	12	Anti-inflammatory, promotes macrophage M2 polarization and angiogenesis	[48]
	Direct current	1 V, Continuous, 100%	30 min	5	Antimicrobial activity, promotes fibroblast proliferation and angiogenesis	[40]
	Direct current	5 V, 4800 Hz, Short-cycle on–off, on–off cycling	60 min	1	Fibroblast proliferation, upregulation of PDGFA, FGF2, TGF-β1	[45]
	Direct current	3 V, Continuous, 100%	30 min	18	Promote cytokine secretion, angiogenesis, and collagen deposition	[46]
Constant current	Pulsed direct current	1–2 V/8 mA, 1 Hz	30 min	10	Anti-inflammatory and macrophage M2 polarization	[38]
	Pulsed direct current	2–6 V/8 mA, 1 Hz	30 min	10	Promoting fibroblast migration and proliferation	[44]
Self-discharge	Direct current	0.5–1.0 V/0.6 mA, Continuous, 100%	Continuous	≥7	Increased ROS for bactericidal action	[42]
	Direct current	0.61 V/0.002 mA, Continuous, 100%	Continuous	14	Promote macrophage M2 polarization, accelerate epithelial migration, and stimulate angiogenesis	[49]
Self-powered	Bipolar pulse	1 V, 1–4 Hz, Millisecond-level, 50%	Several seconds to minutes	7	Hemostatic, antibacterial and promotes fibroblast proliferation	[41]
	Direct current	10 mV, Continuous, 100%	Continuous	15	Reduce ROS, anti-inflammatory, promote angiogenesis, cell proliferation and migration	[43]
	Alternating current	0.1–0.4 μA	Continuous	14	Promote fibroblast migration and proliferation, angiogenesis, and collagen remodeling	[47]
	Alternating current	Mouse: 2.44 V/22.17 nA, Human: 9.64 V/25.21 nA, non-constant,	Continuous	15	Promote cell proliferation and migration, angiogenesis, and collagen deposition	[50]
	Alternating current	0.56 mV –12 V, 2–8 Hz	Continuous	12	Promote fibroblast proliferation and collagen deposition	[51]
	Direct current	0.5–1.5 V, Continuous, 100%	Continuous	6	Inhibits type I collagen and α-SMA, reducing scar area	[52]

#### The hemostatic principle of electrical stimulation physical therapy

As shown in Figure 2e, during the hemostatic phase of wound healing, ES promotes hemostasis through multiple mechanisms. Studies indicate that ES induces intense vasoconstriction in injured vessels, reducing blood flow and minimizing hemorrhage. This process is primarily driven by smooth muscle contraction and may be enhanced by neural signals and humoral mediators such as thromboxane A₂, serotonin, and norepinephrine [34, 93, 94]. Additionally, ES promotes platelet aggregation at the wound site, forming a ‘platelet plug’ while simultaneously activating platelets and inducing the release of platelet-derived growth factor (PDGF), TGF-β, and epidermal growth factor (EGF) [35, 95]. Second, during the secondary hemostasis phase, ES accelerates the cascade reaction of coagulation factors, promoting the driving the transformation of fibrinogen into fibrin. This forms a mesh-like structure that reinforces the thrombus, thereby further arresting bleeding [36]. Concurrently, ES modulates the activity of coagulation factors, increases the production of the thrombin/antithrombin III complex, and shortens bleeding time [37, 95, 96]. Finally, ES can also reduce the release of inflammatory mediators, decrease vascular permeability, and minimize bleeding [97, 98].

#### Anti-inflammatory mechanisms of electrical stimulation therapy

The inflammatory response involves various immune cells—neutrophils, macrophages, and lymphocytes, which respond to the presence of electric fields. During the initial inflammatory window, ES accelerates the recruitment of immune cells and cytokines, promoting wound healing. It attracts neutrophils, macrophages, and lymphocytes to recruit to the injury site, hastening the inflammatory response [38, 39]. As shown in Figure 2f, ES enhances the phagocytic efficiency of macrophages, promotes their M2 polarization, clears bacteria and necrotic tissue from wounds, reduces inflammatory responses, and creates an advantageous setting for wound healing [38, 99]. At the molecular level, ES increases intracellular calcium influx, activates signaling pathways such as AMPK-ERK, and PI3K, elevates ROS levels to exert bactericidal effects, and enhances cellular phagocytosis and migration capabilities [100, 101]. Another study observed that ES switches on pro-regenerative gene programs in both monocytes and macrophages, promoting tissue regeneration [102]. Chronic infection is the primary factor delaying wound healing, while ES disrupts bacterial membrane potential, exhibiting significant antibacterial effects that reduce infection risk, particularly in chronic wounds [40, 41, 103]. Existing studies have demonstrated that ES-mediated regulation of ROS levels exhibits significant parameter dependency and stage specificity. Under high-intensity stimulation (400 mV–5 V), ES upregulates ROS generation during early inflammation by activating NADPH oxidase and the mitochondrial electron transport chain, thereby enhancing phagocytic capacity and migratory activity of neutrophils and macrophages to facilitate pathogen clearance [42, 104–108]. Conversely, during anti-inflammatory and repair phases, low-intensity ES (10 mV–100 mV) suppresses excessive ROS production, induces M2 macrophage polarization, and promotes inflammation resolution, angiogenesis, and collagen deposition to accelerate tissue remodeling [43, 109, 110]. This biphasic ROS regulation precisely matches physiological requirements of distinct healing stages. Ideally, material chemistry should achieve redox balance by integrating ROS-generating units (e.g. TiO₂, Cu^2+^, Fe^2+^) and antioxidant units (e.g. polyphenols, vitamin) within composite scaffolds to simultaneously exert antimicrobial effects and protect host cells [111–114]. In the late stage of inflammation, ES reduces the abundance of immune cells and cytokines, aiding in the resolution of inflammation. It directs the migration of keratinocytes, decreases random motion, and downregulates inflammatory markers of interleukin-8 (IL-8), IL-6, IL-1α, and TNF-α, as well as ROS, thereby accelerating entry into the proliferation phase [38, 97, 98].

#### Proliferation-promoting effects of electrical stimulation therapy

As shown in Figure 2g, during the proliferation phase, ES promotes wound contraction and collagen synthesis by enhancing cell membrane transport and collagen matrix organization. Studies indicate that ES can guide fibroblasts and keratinocytes to migrate and proliferate toward the wound center, accelerating re-epithelialization and wound closure [44, 115, 116]. Urabe *et al.* also reported similar findings, demonstrating that ES enhances the gene expression of platelet-derived growth factors subunit A (PDGFA), TGF-β1, and FGF2, thereby promoting the proliferation of human skin fibroblasts and facilitating wound healing [45]. Concurrently, ES upregulates the level of FGF2, VEGF, C-X-C chemokine receptor type 4 (CXCR4), and other factors through MAPK/ERK signaling pathway and endothelial nitric-oxide synthase (eNOS)-driven NO release. This stimulates endothelial cell proliferation and migration, thereby promoting angiogenesis [46, 47, 99, 117, 118]. Additionally, ES also regulate the level of VEGF, FGF, and epidermal growth factor receptor (EGFR), boosts collagen and elastin production, and enhance the mechanical strength of wounds [119].

#### Electrical stimulation promotes tissue remodeling

As shown in Figure 2h, ES promotes collagen deposition and tissue regeneration, significantly enhances the expression of α-smooth muscle actin (α-SMA) and TGF-β1, facilitates collagen conversion and rearrangement, and strengthens the mechanical integrity of the wound [120, 121]. Importantly, emerging evidence indicates that ES can shift the wound healing paradigm from scar-forming repair toward true regeneration. Zhong *et al.* reported that conductive hydrogels combined with ES amplify endogenous electrophysiological functions and promote macrophage phenotype switch, synergistically realizing scarless wound healing [122]. Furthermore, Yang *et al.* demonstrated rapid and scar-free wound repair using flexible conductive dressings under ES [123], while Fu *et al.* proposed the strategic shift from passive anti-fibrotic to proactive, programmable pro-regenerative approaches for scarless healing [124].

Beyond structural repair, ES demonstrates remarkable potential in regenerating skin appendages. Regarding hair follicle and sebaceous gland regeneration, Yao *et al.* developed a motion-activated wearable ES device that effectively reactivates hair follicle activity and promotes hair regrowth by activating keratinocyte growth factor and VEGF expression, while stimulating keratinocytes within hair follicles and sebaceous glands [125]. Additionally, Yan *et al.* and Hwang *et al.* collectively demonstrated that ES enhances the proliferation and transitional capacity of human dermal papilla cells. Yan *et al.* establishing its significant potential for clinical hair regenerative medicine [126], while Hwang *et al.* corroborated these findings in both human hair follicle-derived papilla cells and animal models using micro-current stimulation [127]. For sweat gland regeneration, which remains one of the most challenging aspects of burn wound healing, Pi *et al.* demonstrated that a flexible sono-piezo patch creates an endogenous regenerative microenvironment through low-intensity pulsed ultrasound (US)-activated ES, achieving functional sweat gland repair in burn injuries [11].

Piezoelectric materials generate piezoelectric stimulation by converting physiological mechanical energy into self-powered endogenous electrical signals, thereby ameliorating scar formation through mediating directional fibroblast migration, promoting orderly collagen alignment, reducing scar width, and modulating fibroblast activity and collagen cross-linking to inhibit excessive fibrosis [122, 123]. Furthermore, when combined with advanced conductive biomaterials, piezoelectric stimulation shows promise in achieving scarless wound healing with complete skin appendage regeneration, resulting in healed tissue that more closely resembles normal tissue both esthetically and functionally [11, 122–124].

### Electrospun piezoelectric biomaterials for skin wound healing

Electrospinning and piezoelectric nanofiber technology, which integrates the piezoelectric effect with the electrospinning process, provides an innovative material platform for skin wound healing. While various spinning technologies can process polymers, ceramics, or metals into fibrous forms, including melt spinning, solution spinning, air-jet spinning, template synthesis, and self-assembly/phase separation [128], electrospinning offers a superior balance in the fabrication of nanoscale fibers. Compared to other techniques, electrospinning is highly efficient, stable, and controllable. The process inherently subjects the polymer jet to high-voltage electrostatic forces and mechanical stretching, which facilitates the orientation of molecular chains and the formation of piezoelectric phases. The resulting nanofibrous structures possess a high specific surface area, high porosity, and ease of functionalization. These characteristics endow the materials with significant potential in diverse fields, particularly in biomedical applications, environmental governance, energy storage, and smart textiles [129].

It is evident that, in accordance with their source and composition, electrospun piezoelectric biomaterials can be classified into four primary categories: natural polymers, synthetic polymers, inorganic materials, and their composites. Each material class offers distinct advantages in terms of piezoelectric performance, biocompatibility, and functional integration, collectively driving the technology's translation toward clinical application. However, the sector faces significant challenges, including the optimization of performance and the validation of safety.

#### Natural polymer electrospun piezoelectric materials

Electrospinning of natural organic piezoelectric materials demonstrates significant potential in the field of wound healing. The primary benefits of these materials are attributable to their exceptional biocompatibility, biodegradability, and inherent bioactivity. These properties enable the creation of a microenvironment that closely mimics the natural ECM, exhibiting superior compatibility with human tissues and significantly reducing the risk of immune rejection and inflammatory responses [130–132].

**Figure 3:**
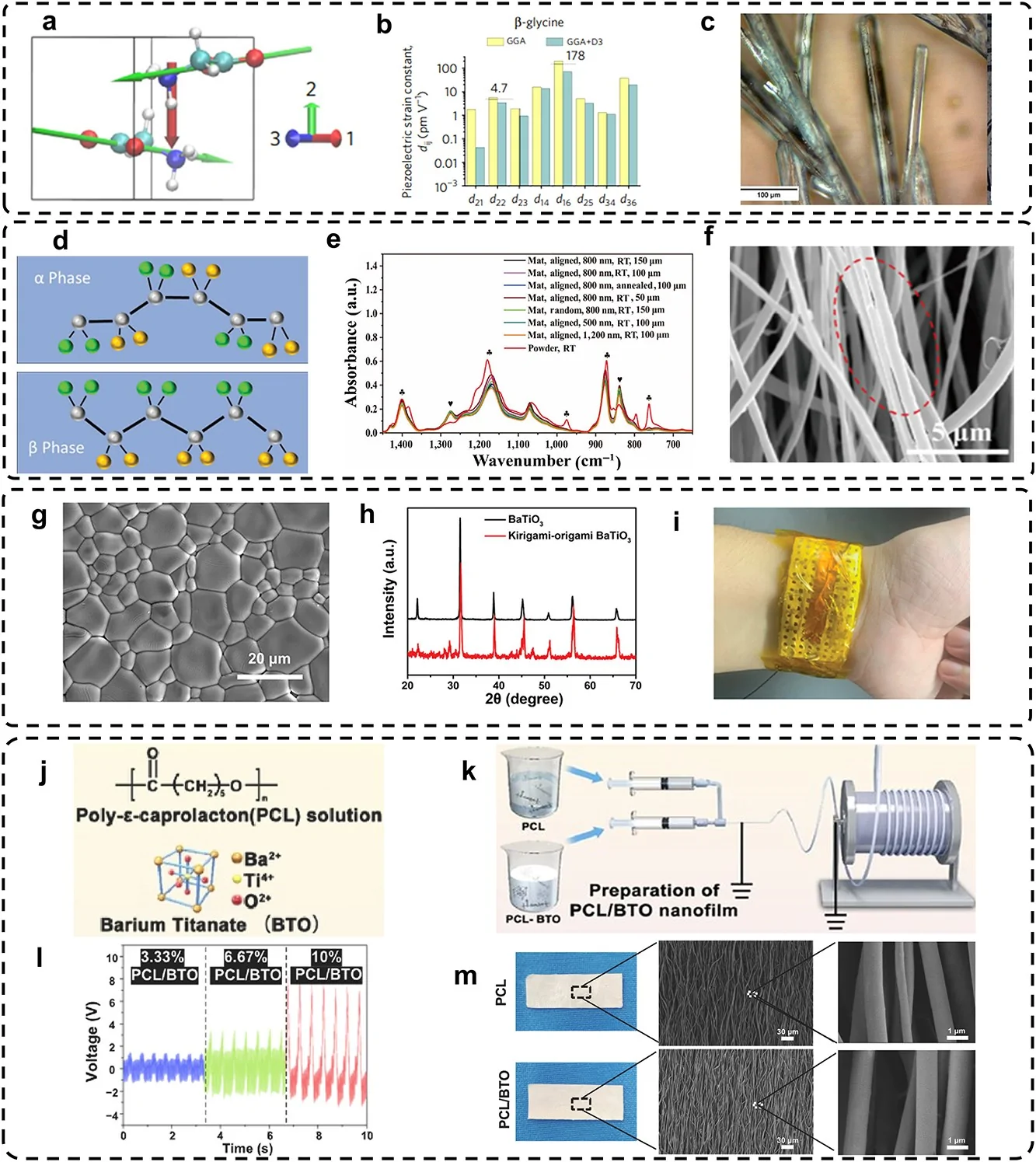
Electrospun piezoelectric materials for skin wound healing. (**a**) The molecular structure of β-glycine. Reproduced with permission [20]. Copyright 2018, Springer Nature. (**b**) The piezoelectric coefficients of β-glycine. Reproduced with permission [20]. Copyright 2018, Springer Nature. (**c**) The microscopic image of β-glycine needles. Reproduced with permission [20]. Copyright 2018, Springer Nature. (**d**) The molecular structure of the PVDF polymer. Reproduced with permission [144]. Copyright 2018, John Wiley and Sons. (**e**) FTIR spectra of different PVDF materials. Reproduced with permission [22]. Copyright 2020, Springer Nature. (**f**) PVDF nanofiber membrane. Reproduced with permission [22]. Copyright 2020, Springer Nature. (**g**) SEM image of BTO. Reproduced with permission [24]. Copyright 2023, John Wiley and Sons. (**h**) The piezoelectric strength of BTO. Reproduced with permission [24]. Copyright 2023, John Wiley and Sons. (**i**) Image of the wound dressing. Reproduced with permission [24]. Copyright 2023, John Wiley and Sons. (**j**) Molecular formula of PCL and structural formula of BTO. Reproduced with permission [160]. Copyright 2025, KeAi. (**k**) The fabrication process of the PCL/BTO nanofiber membrane. Reproduced with permission [160]. Copyright 2025, KeAi. (**l**) The piezoelectric coefficients of PCL/BTO. Reproduced with permission [160]. Copyright 2025, KeAi. (**m**) SEM images of the PCL and PCL/BTO nanofiber membranes. Reproduced with permission [159]. Copyright 2025, KeAi

Amino acids are the fundamental building blocks of proteins. Among them, glycine exhibits considerable piezoelectric properties due to its unique molecular structure (Fig. 3a) [20]. Glycine crystals are known to occur in three distinct phases: α, β, and γ. The γ-glycine crystal is characterized by a non-centrosymmetric space group structure, wherein its helical hydrogen bond network gives rise to a net dipole moment, resulting in a piezoelectric coefficient d₃₃ of ~10.4 pm/V (Fig. 3b and c) [20, 133, 134]. Protein-based biomaterials are extensively employed in piezoelectric electrospinning, with silk fibroin, collagen, keratin, and gelatin being the primary constituents [135]. These proteins are characterized by a high concentration of amide bonds (-CONH-) and polar groups, which facilitates the generation of a piezoelectric effect through the ordered arrangement of their molecular chains [134]. Polysaccharide materials are also widely applied in piezoelectric electrospinning. Their molecular chains contain numerous polar groups, such as hydroxyl (-OH), amino (-NH_2_), and carboxyl (-COOH) groups, which have the capacity to generate a piezoelectric effect when present in crystalline regions or when orderly arranged [136, 137]. Among these, cellulose is one of nature's most piezoelectric polysaccharides, and its Iβ crystal form can exhibit a piezoelectric coefficient of 36.4 pm/V [138–140].

#### Electrospun piezoelectric materials from synthetic polymers

Electrospinning of synthetic polymer piezoelectric materials offers a highly tunable structure and superior processing properties. The chemical structure, molecular weight, hydrophilicity/hydrophobicity, degradation rate, and mechanical properties of these materials can be modulated to meet the requirements of different tissue repair applications. Furthermore, the synthesis process is highly controllable, which facilitates large-scale production [141–143].

Polyvinylidene fluoride (PVDF) and its copolymers possess the highest piezoelectric coefficients and are currently the most extensively studied piezoelectric polymers. The piezoelectricity exhibited by these materials is predominantly attributed to the β-crystal phase (Fig. 3d) [144]. The piezoelectric coefficient d₃₃ of pure PVDF is ~20–28 pC/N (Fig. 3e and f) [145–147]. The piezoelectric coefficient d₃₃ of electrospun poly (l-lactic acid) (PLLA) has been shown to reach 19 pC/N, piezoelectric constant d_14_ of ~10 pC/N and the material is also biodegradable [148–150]. The piezoelectric properties of polyurethane (PU) are attributable to the presence of urea-based dipoles within its molecular structure. The piezoelectric performance of PU has been shown to diminish as the carbon chain of the diamine lengthens, a phenomenon that is closely linked to the reduction in urea-based dipole density [151].

#### Electrospun inorganic-based piezoelectric materials

Inorganic ceramic piezoelectric materials, including lead zirconate titanate (PZT), barium titanate (BTO), zinc oxide (ZnO), and aluminum nitride (AlN), have been identified as having significant potential for utilization in wound repair applications, owing to their exceptional piezoelectric properties [152, 153]. The primary advantage of these materials lies in their piezoelectric coefficients, which are significantly higher than those of organic materials. This facilitates the effective conversion of weak physiological mechanical signals into potent ES, thereby significantly promoting cell behavior and tissue regeneration. The piezoelectric effect in inorganic ceramics is attributable to their non-centrosymmetric crystal structures. When subjected to an external force, the centers of positive and negative charges within the unit cell shift relative to each other, forming a macroscopic electric dipole moment and generating a piezoelectric response [154].

BTO is a typical perovskite-structured ferroelectric ceramic (Fig. 3g) [24]. It has been demonstrated that the piezoelectric coefficient d₃₃ of the material can exceed 190 pC/N (Fig. 3h), which is significantly higher than that of most polymeric materials [24, 155]. In the context of wound repair applications, BTO is conventionally incorporated as nanoparticles and composited with biodegradable polymers such as poly(lactic-co-glycolic acid) (PLGA) and polyvinyl alcohol (PVA) to fabricate composite nanofiber scaffolds via electrospinning technology (Fig. 3i) [24]. Among piezoelectric ceramics, PZT is one of the most prominent, exhibiting a piezoelectric coefficient (d₃₃) of up to several hundred picocoulombs per newton, which is approximately two to three times higher than that of BTO [156, 157]. Notwithstanding its remarkably robust piezoelectric response, PZT contains lead, which introduces a potential biotoxicity hazard. This significantly limits its application in long-term implantable medical devices [158]. ZnO is a multifunctional material that combines piezoelectricity, antibacterial properties, and good biocompatibility, and as such has attracted significant attention in the field of wound repair. The piezoelectric coefficient of the material, d₃₃, is ~12–15 pm/V [152]. However, its unique antibacterial properties offer an ideal solution for chronic wounds with a high risk of infection.

#### Electrospun composite piezoelectric materials

Electrospinning of composite piezoelectric materials demonstrates immense potential by integrating the advantages of different materials, offering significant benefits in tuning piezoelectric performance, structural design flexibility, and multifunctional integration. This approach has emerged as a pivotal strategy for overcoming the performance limitations inherent to single-component materials [159]. In the preparation of composite scaffolds, particularly when incorporating inorganic piezoelectric fillers into polymers, achieving a homogeneous distribution of fillers is a critical prerequisite for ensuring consistent performance. Agglomeration of fillers can lead to stress concentration and non-uniform electrical output. To accomplish this objective, several effective strategies are employed. Surface modification using silane coupling agents or surfactants is widely utilized to enhance the compatibility between fillers and the polymer matrix, thereby reducing interfacial tension [160]. In parallel, physical dispersion techniques such as probe ultrasonication combined with prolonged magnetic stirring are essential for breaking up aggregates within the precursor solution [161]. Furthermore, optimizing solution properties, specifically viscosity and conductivity, helps to stabilize the suspension and prevent sedimentation during the electrospinning process [162].

Advancements in electrospinning technology and the emergence of eco-friendly fabrication processes have led to a proliferation of potential applications for piezoelectric composites in the domain of wound repair. In the context of skin wound healing, piezoelectric composites have been shown to facilitate efficient healing and functionalization through the integration of piezoelectric polymers with inorganic ceramics or natural polymers [108]. For instance, a PVDF@titanium dioxide (TiO_2_) composite has been shown to generate a significant piezoelectric voltage output when subjected to an external force. This ES has been demonstrated to effectively promote fibroblast proliferation and collagen synthesis, thereby accelerating skin wound healing (Fig. 3j) [159]. In the context of complex diabetic wounds, the utilization of chitosan/PVA/ZnO nanofiber membranes has been demonstrated to result in a substantial acceleration of the healing process. Their excellent antibacterial properties and piezoelectric effect work synergistically, providing an ideal solution for diabetic wound dressings (Fig. 3k) [159, 163]. Furthermore, by compositing PVA with natural polymers such as sodium alginate, chitosan, and gelatin, the scaffold's water absorption capacity, as well as its cell adhesion and proliferation properties, can be significantly enhanced, further optimizing the wound healing microenvironment (Fig. 3l and m) [159, 164].

#### Advantages and disadvantages of various material types

In the development of electrospun piezoelectric materials, each material system exhibits unique advantages while also facing corresponding challenges. It is evident that natural piezoelectric biomaterials exhibit both good biocompatibility and biodegradability. However, these materials are inherently limited by factors such as their weak piezoelectric performance, their poor processability, and their low mechanical strength. In order to enhance performance, a range of strategies must be considered, including the optimization of molecular orientation, the control of crystallinity, and the reinforcement of composites. Synthetic polymer materials have been demonstrated to exhibit excellence in controllable processing and piezoelectric response. Nevertheless, concerns remain regarding their insufficient bioactivity and the potential for their degradation products to induce inflammation. However, further research is required to comprehensively evaluate their long-term biocompatibility and potential toxicity. Despite the fact that inorganic piezoelectric materials exhibit excellent piezoelectric properties, they are confronted with challenges such as bio-inertness, complexity in degradation processes, and a disparity between the mechanical properties of these materials and those of biological tissues. Moreover, the high brittleness of these materials and the difficulties often associated with their processing frequently necessitate their incorporation within composite polymer materials. While composite piezoelectric materials have the capacity to integrate the advantages of multiple material types, the majority of current research remains at the laboratory stage. These challenges are attributed to three main factors: firstly, the complexity of the fabrication processes involved; secondly, the significant financial costs associated with the development process; and thirdly, the paucity of large-scale clinical validation. In the future, there is an urgent need to advance standardized, scalable fabrication technologies and to conduct systematic preclinical and clinical studies (Table 2) [165–184].

**Table 2:** Performance of different electrospun piezoelectric materials

Material category	Representative material	Piezoelectric properties	Biocompatibility	Workability	Mechanical strength	Degradability	Limitation	Ref.
Natural polymer material	Cellulose	Iβ: 36.4 pm/V	Excellent (low immunogenicity)	Poor (process optimization is necessary)	Lower	Degradable	Poor piezoelectric performance, poor processability, low mechanical strength.	[133, 135, 139, 140, 165–170]
	Glycine	γ: 10.4 pm/V						
	Silk fibroin	1.5 pC/N						
	Collagen	0.1–1 pC/N						
Synthetic polymer material	PVDF	20–28 pC/N	Average (potential for inflammation)	Excellent (adjustable)	Rather high	PVDF: non-degradable; PLLA: degradable; PU: degradable;	Insufficient biological activity, degradation by products may induce inflammation, long-term toxicity requires further evaluation.	[145, 146, 149, 171–177]
	PLLA	d_33_: 19 pC/N						
		d_14_: ~10pC/N						
	PU	1–40 pC/N						
Inorganic materials	BTO	>190 pC/N；	Poor	Poor (requires combined machining)	High strength but very brittle	Non-degradable	Long-term foreign body risk, difficulty in processing, modulus mismatch with tissue, lead toxicity of PZT.	[155–157, 178–183]
	PZT	200–600 pC/N						
	ZnO	12–15 pC/N						
Complex materials	PCL/BTO	35–45 pC/N	Good (synergistic optimization)	Average (complex process)	High (composite reinforcement)	Regulated	High production cost, lack of clinical data, challenges in scaling up production.	[159, 163, 164, 184]
	Chitosan/	15–25 pC/N						
	PVA/ZnO							
	PVDF/	30–45 pC/N						
	Hydroxyapatite							

### Structures and fabrication of electrospun piezoelectric materials

Electrospinning technology is a process which utilizes a unique mechanism driven by electric field forces to efficiently fabricate fibrous materials from the nanoscale to the microscale. The classification of the technology is dependent on the spatial arrangement and structural dimensionality of the nanofibers, and can therefore be divided into three major structural systems: one-dimensional (1D) (sutures), two-dimensional (2D) (membrane), and three-dimensional (3D) (scaffolds). Through precise control over process parameters and material composition, these electrospun structures of different dimensions exhibit distinct morphological features and performance advantages. In the biomedical field, particularly in relation to wound healing, tissue regeneration and repair, they demonstrate broad application potential and unique value, ranging from fundamental suturing and wound coverage to the construction of complex scaffolds.

#### 1D structures: electrospun sutures

**Figure 4:**
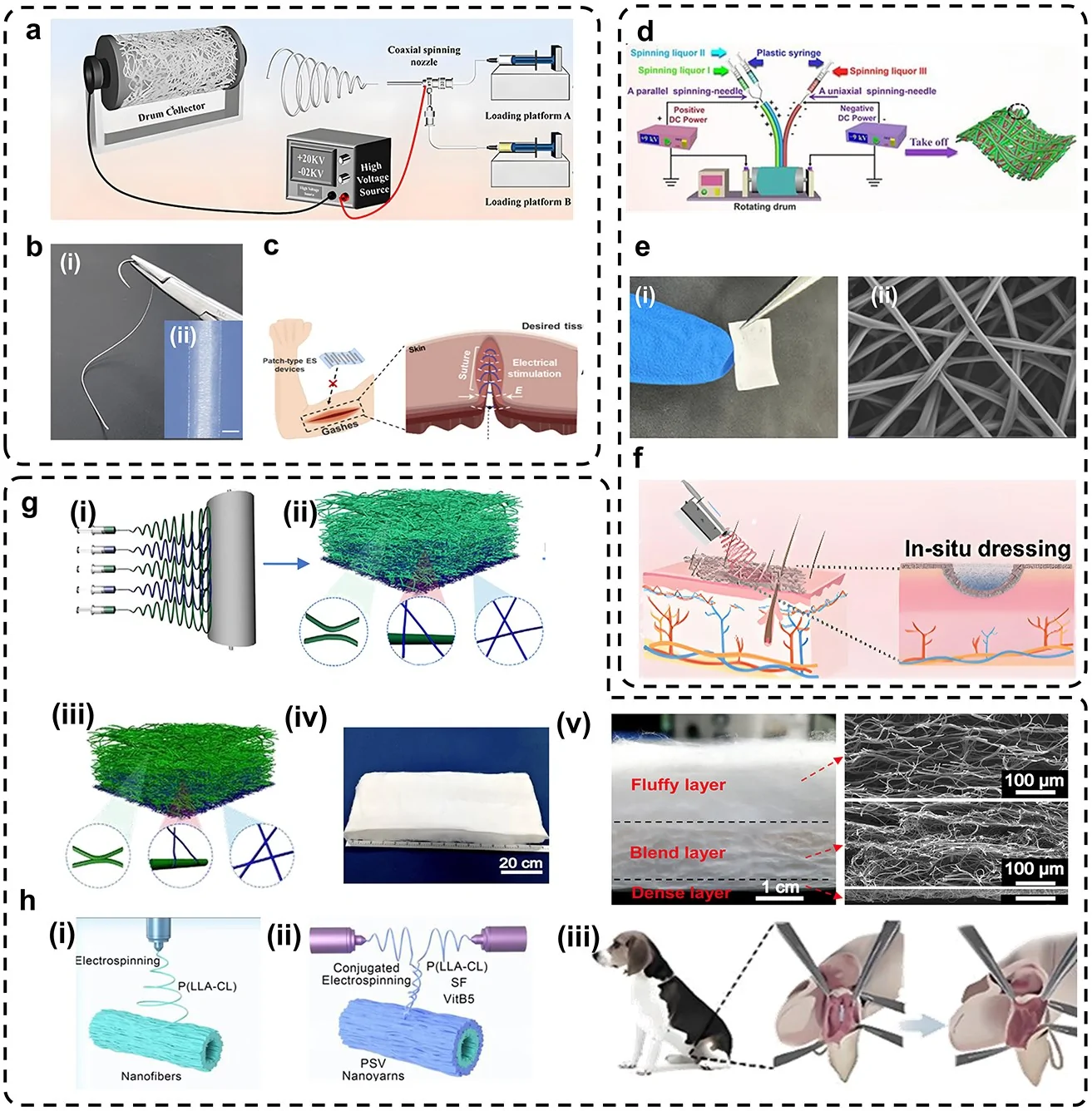
Structures and fabrication of electrospun piezoelectric materials. (**a**) Formation of the 1D electrospun nanofiber suture. Reproduced with permission [185]. Copyright 2025, Elsevier. (**b**) Image of 1D electrospun nanofiber suture. Reproduced with permission [190]. Copyright 2025, Elsevier. (**c**) Application of the nanofiber suture for wound repair. Reproduced with permission [190]. Copyright 2025, Elsevier. (**d**) Formation of 2D electrospun nanofiber membrane. Reproduced with permission [191]. Copyright 2025, Elsevier. (**e**) Schematic of 2D electrospun nanofiber membrane (i). SEM image of an e nanofiber membrane (ii). Reproduced with permission [193]. Copyright 2024, Springer nature. Reproduced with permission [195]. Copyright 2025, Springer nature. (**f**) Schematic of the nanofiber membrane used for skin wound healing. Reproduced with permission [196]. Copyright 2024, Springer nature. (**g**) Formation of 3D electrospun nanofiber structures (i—iii). Image of 3D electrospun nanofiber structures (iv). Optical and SEM images of 3D nanofiber structures (v). Reproduced with permission [197]. Copyright 2021, Elsevier. (**h**) Formation of 3D electrospun nanofiber scaffold (i, ii). The scaffold for canine urethral repair (iii). Reproduced with permission [198]. Copyright 2023, Springer nature

1D electrospinning technology is a process that utilizes a high-voltage electrostatic field to draw a polymer solution or melt into continuous nano- to micro-fiber bundles or yarns with sufficient strength and weave compatibility. In the presence of a high electric field, a polymer droplet has been observed to overcome surface tension, resulting in the formation of a jet. As the jet travels, the solvent evaporates or the melt cools, ultimately solidifying into a 1D nanofiber that is deposited on a collector (Fig. 4a) [185]. The morphology of a typical 1D electrospun nanofiber is linear, with an aspect ratio that is extremely high. In addition to providing ES functionality when made from piezoelectric materials, the yarns possess an extremely high specific surface area and porosity due to their nano- to micro-scale diameter, facilitating drug loading, cell adhesion, and mass exchange [186, 187]. It is evident that the tensile strength and toughness of the yarn can be significantly enhanced through various processes, including twisting, orientation, and composite reinforcement. This enhancement is crucial for meeting the stringent requirements for implantable applications.

At the microstructural level, 1D electrospun nanofibers have been observed to form various single-fiber structures, including core-shell and beaded nanofibers [188]. The fabrication of core-shell nanofibers is predominantly accomplished through the utilization of a coaxial electrospinning process, wherein two solution streams converge at the spinneret's exit to form a compound Taylor cone. This cone is subsequently ejected as a coaxial jet under the influence of a high-voltage electric field [189]. The core-shell structure of the nanofiber enables the sustained release of the drug in the context of drug delivery applications. Additionally, coaxial electrospinning has been demonstrated as a viable method for the production of nanofibers from polymers that are not readily amenable to direct spinning via conventional methodologies. The formation of beaded nanofibers is attributable to axial instability experienced by the fluid jet during the electrospinning process. Factors such as altering solution concentration and viscosity, increasing surface tension, changing the electric field strength, or increasing the collection distance typically leads to bead formation. While initially regarded as a process defect, recent studies have demonstrated that beaded structures can be utilized to effectively modulate the nanofiber's surface roughness.

In the context of clinical applications, the repair of skin and muscle tissue constitutes a critical aspect of postoperative treatment, wherein suturing technology plays a pivotal role. Electrospun sutures with ES functionality have been shown to provide local ES, as well as effectively promoting tissue regeneration, ECM deposition, and vascularization, thereby accelerating the wound healing process (Fig. 4b and c) [190]. Moreover, as an absorbable material, it can gradually degrade and be absorbed by the body during the tissue recovery process, thus eliminating the need for a second surgery for removal. This is a key advantage of minimally invasive therapy.

#### 2D structures: electrospun membrane

2D electrospinning technology is employed to create a thin-film structure, a process which involves modifying the collector and the electric field setup in order to enable the ordered deposition of nanofibers on a 2D plane. The fundamental principle of this process involves the establishment of an electrostatic field between two parallel electrodes. When a polymer solution or melt is introduced into this field, it is drawn by the electrostatic force to form nanofibers, which are then deposited on a collector to form a continuous 2D fibrous membrane (Fig. 4d) [191]. It is evident that 2D electrospinning confers numerous advantages, including a highly controllable structure, an enlarged specific surface area, tunable porosity, and excellent biocompatibility [192]. Through the precise manipulation of the nanofiber orientation, diameter, and composition, the physical properties and biological functions of the membrane can be meticulously designed, thereby providing a highly customizable material platform for biomedical applications. In comparison with conventional medical gauze, 2D electrospun structures based on piezoelectric materials provide local ES to promote healing. In addition, they offer superior coverage and greater affinity for irregular wound surfaces, thereby promoting blood clotting and accelerating the absorption of exudate. Depending on the nanofiber arrangement, two main fibrous structures can be formed by 2D electrospinning: randomly oriented nanofibers and aligned nanofibers. The most prevalent form of deposition is random nanofibers, which are obtained using a conventional grounded conductive plate collector (Fig. 4e) [193–195]. The alignment of nanofibers is predominantly accomplished through the modification of collector designs, such as rotating drums or parallel plate electrodes. Furthermore, the introduction of external force fields, such as magnetism, can be utilized to direct fiber alignment. In addition, it is imperative to exercise precise control over the spinning process, as this is instrumental in optimizing parameters and thereby governing the trajectory of the charged jet, thereby suppressing its inherent bending instability.

Nanofibrous membranes prepared by 2D electrospinning hold significant application value in the biomedical field, being particularly suitable as dressings for chronic wounds (Fig. 4f) [196]. The structural features of these materials have been demonstrated to facilitate the absorption of wound exudate and gas exchange, thereby effectively impeding bacterial invasion and establishing an optimal microenvironment for wound healing. It is evident that the flexible structural control capabilities and excellent functional integration of 2D electrospinning technology have led to its emergence as a core method for constructing advanced wound dressings and tissue engineering scaffolds. There is considerable clinical potential in the utilization of this technology to promote the efficient healing of complex wounds and tissue regeneration.

#### 3D structures: electrospun scaffolds

3D electrospinning technology is a process which involves the construction of a 3D, structured nanofiber network, which is formed directly on the collector. The fundamental mechanism of this process entails the utilization of multi-nozzle systems or dynamic electric field regulation devices, which induce the formation of multiple jets within the electric field. The deposition of nanofibers leads to the formation of a 3D, ordered accumulation (Fig. 4g) [197]. It is evident that 3D electrospun structures exhibit considerable advantages in addressing the challenges associated with the repair of deep, large-volume, and irregularly shaped wounds. This is primarily due to their biomimetic 3D spatial framework and efficient mass exchange properties. Their structural characteristics provide a 3D space for cell proliferation and mechanical support. In addition, through high porosity and interconnectivity, they promote nutrient transport and metabolic waste removal, thus creating an ideal microenvironment for tissue regeneration.

The construction of the macroscopic structure of 3D electrospun nanofibers is primarily informed by the following four strategies. The initial approach involves the extension of the spinning duration to establish a multi-layered, stacked nanofiber structure through continuous deposition. The second stage is the post-processing assembly of 2D nanofiber mats, where pre-prepared 2D fibrous membranes are integrated into a 3D structure through stacking, folding, or lamination techniques. Another approach is template-assisted direct assembly, which uses preset 3D templates, such as micropillar arrays or spherical scaffolds, to guide directional nanofiber deposition. Finally, the process of self-assembly is worthy of note. In this method, nanofibers spontaneously form an interconnected 3D network during deposition by optimizing parameters such as the electric field and nozzle direction. For instance, the employment of a humidity-assisted multi-step electrospinning process in conjunction with a physico-chemical dual-crosslinking method facilitates the fabrication of a gradient-structured nanofiber sponge. The construction of a Z-direction gradient with a porous layer, a semi-porous layer, and a dense layer has been demonstrated to overcome the limitations of 2D nanofiber membranes, thereby achieving a combination of ultra-lightweight, high elasticity, and hydrophobicity.

The 3D nanofiber networks prepared by 3D electrospinning have considerable potential for application in the biomedical field. Research has demonstrated that a bilayer nanofiber sponge tube, fabricated using two electrospinning techniques, has been shown to successfully achieve scar-free urethral regeneration (Fig. 4h). In this construct, the inner layer of dense nanofibers effectively prevents leakage, while the outer layer features a 3D sponge-like architecture composed of silk fibroin and Vitamin B5. This design creates a synergistic effect: the incorporation of Vitamin B5 imparts intrinsic hydrophilicity, whereas silk fibroin contributes to mechanical flexibility and toughness; simultaneously, the porous framework physically augments elasticity and liquid absorption capacity [198].

#### Microstructures of piezoelectric electrospun materials

Piezoelectric electrospinning enables the fabrication of three distinct microstructures through the adjustment of collection devices and process parameters.

Randomly oriented structures are formed using a static flat plate collector, where fibers are chaotically intertwined into a non-woven-like mesh mat [199, 200]. The manufacturing process for this structure is simple and easily scalable; its uniform properties in all directions make it suitable for flexible sensors and filtration materials [27, 201]. However, the chaotic fiber direction leads to inconsistent internal piezoelectric dipole orientations, resulting in low macroscopic piezoelectric output efficiency. Additionally, the numerous contact points cause significant mechanical energy transmission loss, and sensitivity is typically lower than that of aligned structures [129, 202].

Aligned structures are commonly achieved via high-speed rotating drum collection or parallel electrode collection methods. Under the influence of electric field forces or mechanical drawing forces, fibers extend highly orderly in a single direction to form parallel fiber arrays [203]. The high-speed drawing action improves the orientation of polymer molecular chains and piezoelectric crystal phases within the fibers, thereby significantly enhancing the axial piezoelectric coefficient. The structure exhibits obvious anisotropy and high mechanical strength, making it suitable for high-performance sensors sensitive to stress in specific directions [204]. However, the preparation process requires stringent control, and collection speed is limited. The bonding force perpendicular to the fiber axis is weak, making the structure prone to delamination; furthermore, the sensing response is directionally dependent, rendering it unable to cope with multi-axial complex strain detection.

Hierarchical structures represent a complex composite morphology containing graded structures of nanofibers and microfibers, nanonode network structures, or hierarchical structures of micro-nano pores on fiber surfaces [205, 206]. The extremely large specific surface area and complex pore structure can effectively reduce the material's bending stiffness, enabling significant deformation under minute external forces, thereby substantially enhancing piezoelectric response sensitivity [200, 207]. This structure is also conducive to cell adhesion or multi-modal signal response. However, the mechanism of structure formation is complex and often influenced by multiple factors such as solvent evaporation rate and environmental humidity, resulting in poor preparation reproducibility. Moreover, the excessively fine secondary structures lack sufficient mechanical stability and are prone to fracture or structural collapse during long-term repetitive deformation [208, 209].

#### Comparison of electrospun structures

1D electrospinning technology demonstrates unique advantages in the field of wound management, endowing materials with multifaceted functions including antibacterial, anti-inflammatory, pro-regenerative, and anti-adhesion properties. The nanofibers produced have the capacity to attain nanoscale diameters and possess a high specific surface area, thereby facilitating the processes of drug loading and release. Moreover, the process is uncomplicated, thus facilitating the attainment of high-purity nanofiber fabrication. However, due to their reduced mechanical strength and the absence of effective inter-nanofiber connections, 1D nanofibers are susceptible to fracture and are challenging to utilize as standalone tissue engineering scaffolds. The development of 1D biodegradable piezoelectric sutures is impeded by a key challenge: these devices typically require substantial mechanical deformation to achieve a useful electrical output. However, the majority of biodegradable materials are inherently brittle. The combination of high flexibility demands and intrinsic material brittleness renders their fabrication at the 1D scale particularly challenging.

The primary benefit of 2D electrospinning is the fabrication of large-area, continuous nanofiber membranes. These membranes are distinguished by their high porosity, tunable pore size and thickness, which facilitate gas exchange and fluid transport. Additionally, they exhibit excellent flexibility, enabling them to conform closely to irregular wound surfaces. Through the precise regulation of parameters such as voltage, intensity, spinning distance, and solution flow rate, the diameter, density, and thickness of the nanofibers can be effectively managed to yield an ordered nanofiber network with specific mechanical properties and biocompatibility. However, the limited thickness and dense arrangement of 1D and 2D nanofibers offer restricted pathways for cell migration and inefficient nutrient permeation, severely constraining 3D cell proliferation and tissue integration.

In contrast, 3D electrospun scaffolds effectively overcome the aforementioned limitations by constructing a high specific surface area, an interconnected porous structure, and a biomimetic ECM microenvironment. This 3D architecture has been demonstrated to significantly promote cell migration, differentiation, and tissue regeneration during long-term culture, thereby providing an indispensable 3D space for cell proliferation and vascularization (Table 3).

**Table 3:** Summary of properties of electrospun structures with different dimensions

Electrospun structure	Principle	Structural types	Application fields	Advantages	Limitations	Innovative applications	Ref.
1D structure —electrospun sutures	Based on electrostatic forces, a high-voltage electric field ejects and stretches a polymer solution into a single filament, which is deposited on a collector to form a monofilament structure.	Core-shell nanofibers, beaded nanofibers, porous nanofibers, etc.	Drug carriers, drug delivery, tissue engineering, wound dressings, surgical sutures.	Nanoscale diameter; high specific surface area; facilitates drug loading and release; simple process; can possess multiple functions such as antibacterial, anti-inflammatory, pro-regenerative, and anti-adhesion properties; biodegradable.	Low mechanical strength; lack of inter-nanofiber connections; prone to breakage; difficult to use alone as a tissue engineering scaffold.	Electro-stimulating sutures: provide ES to promote tissue regeneration, extracellular matrix deposition, and vascularization, thereby accelerating wound repair.	[185–190]
2D structure — electrospun membrane	By modifying the collection device and electric field settings, nanofibers are deposited in an orderly manner on a 2D plane to form a film/membrane structure.	Randomly oriented nanofibers, aligned nanofibers, etc.	Chronic wound dressings (e.g. for diabetic foot ulcers), wound healing.	Large-area continuous film; high porosity with tunable pore size and thickness; excellent flexibility to conform to the wound; promotes hemostasis and exudate absorption; acts as a bacterial barrier, providing an ideal healing environment.	Limited thickness; densely packed nanofibers restrict pathways for cell migration and result in low nutrient permeation efficiency.	Personalized nanofiber dressings: provide customized treatments for different wound types.	[192–194, 196]
3D structure—electrospun scaffolds	By precisely controlling the spatial distribution and 3D stacking of nanofibers, a nanofiber network with a 3D structure is constructed.	Gradient-structured nanofiber sponges, bilayer nanofiber sponge tubes, etc.	Sophisticated bio-scaffolds, bone tissue engineering, scar-free urethral regeneration, trauma repair.	Nanoscale resolution and high precision; capability for constructing complex structures; provides a 3D space to promote cell proliferation; high specific surface area and interconnected porous structure.	Relatively complex fabrication process; requires precise control of multiple parameters.	Biomimetic bilayer scaffold: achieves scar-free urethral regeneration; gradient-structured nanofiber sponge: achieves a unified combination of ultra-lightweight, high elasticity, and hydrophobicity.	[197, 198]

### Application of piezoelectric electrospinning in wound healing

Electrospun piezoelectric biomaterials have been shown to undergo electrical polarization when subjected to mechanical stress, such as stretching, ultrasonic vibration, or the influence of a magnetic field. This process leads to the generation of a potential difference on the material's surface, thereby achieving the conversion of mechanical energy into electrical signals. The primary advantage of this system is attributed to a ‘passively activated’ intelligent therapeutic mechanism, which functions independently of an external power source. Rather, it relies on movement at the wound site to trigger ES, thereby achieving dual regulation in both infection control and tissue regeneration [210, 211]. Research has indicated that piezoelectric biomaterials have considerable potential for application in the domain of wound repair [212]. Fundamentally, piezoelectric electrospun materials are nanofibers fabricated via electrospinning technology that possess piezoelectric properties. This technology utilizes high-voltage electrostatic forces to process polymer solutions or melts into nano- or micro-scale fibers [192, 213]. The resulting materials not only enable the interconversion of mechanical and electrical energy but also retain the inherent advantages of nanofibers, such as being lightweight, flexible, and having a high specific surface area [14, 15, 214]. By converting mechanical energy into electrical energy, these materials create a favorable microenvironment to accelerate wound healing. To provide a systematic review, the following sections will discuss the applications of piezoelectric electrospun materials in wound healing based on their activation mechanisms and functional characteristics. The discussion is divided into three subsections: Motion-Induced Electrical Stimulation for Wound Healing, Non-Motion-Activated Electrical Stimulation for Wound Healing, and Multifunctional Synergistic Piezoelectric Materials.

#### Motion-induced electrical stimulation for wound healing

**Figure 5:**
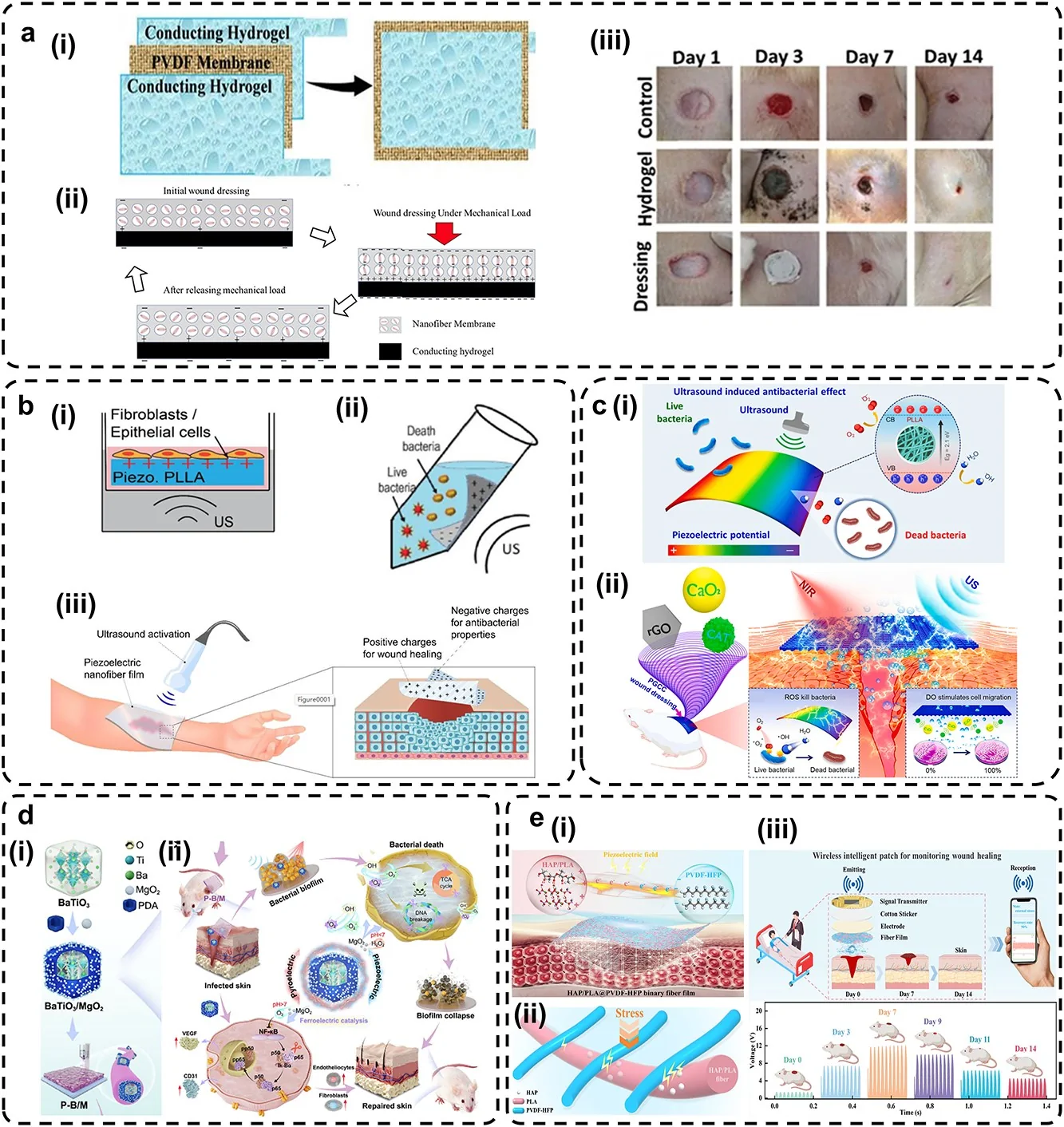
Application of piezoelectric nanofiber in wound healing. (**a**) Schematic diagram of the piezoelectric nanofibers and electroactive hydrogel composite dressing (i). Charge generation mechanism (ii). Images of wounds on days 1, 3, 7, and 14 (iii). Reproduced with permission [215]. Copyright 2022, Elsevier. (**b**) Schematic of US-activated scaffold (i). Schematic of US-activated nanofiber treatment for bacteria (ii). Schematic of piezoelectric nanofibers on a wound (iii). Reproduced with permission [54]. Copyright 2023, Elsevier. (**c**) Schematic of piezocatalytic ROS generation by PGCC (i). Schematic of accelerated healing of infected wounds (ii). Reproduced with permission [112]. Copyright 2023, Elsevier. (**d**) Preparation of the dual-catalytic membrane (i). Antibacterial, anti-inflammatory and pro-regenerative properties of P-B/M membranes (ii). Reproduced with permission [220]. Copyright 2025, John Wiley and Sons. (**e**) Schematic of the wound healing effect of the HAP/PLA@PVDF–HFP nanofiber membrane(i). Multiple modes of the HAP/PLA@PVDF–HFP nanofiber membrane (ii). Schematic of a wireless smart patch for monitoring wound healing (iii). Reproduced with permission [221]. Copyright 2025, Elsevier

As illustrated in Figure 5a, a composite dressing has been developed which combines an electrospun PVDF piezoelectric film with a conductive hydrogel composed of carbonized polydopamine (PDA)/polyacrylamide [215]. This dressing has been demonstrated to convert mechanical movement into ES, thereby synergizing with the moist healing environment to significantly promote the repair of refractory wounds, including diabetic foot ulcers. The output of a direct current ES of 42 mV and 60 nA at a frequency of ~5 Hz effectively accelerates fibroblast proliferation and the directional migration of endothelial cells, thereby promoting angiogenesis, tissue remodeling, and wound healing, achieving a 100% wound healing rate by day 14. Concurrently, the hydrogel component absorbs exudate and maintains a moist environment, while the PDA endows the material with contact-killing capabilities, thereby reducing the risk of infection. The development of a self-powered, multifunctional wound dressing, offering a novel strategy for the treatment of chronic wounds, was achieved through the synergistic design of a piezoelectric-triboelectric nanogenerator and a conductive hydrogel.

#### Non-motion-activated electrical stimulation for wound healing

When activated by external energy sources, electrospun piezoelectric materials have the capacity to provide non-contact ES therapy [54, 216]. Figure 5b shows a tissue engineering strategy employing a biodegradable, self-charging PLLA nanofiber scaffold has been demonstrated. When activated by US, the scaffold generates a direct current signal (300 mV) that facilitates wound healing and skin regeneration through several mechanisms, including the production of ROS, the regulation of cell proliferation and gene expression, and the modulation of the immune response. As demonstrated in the pertinent animal studies, the treatment of wounds with this scaffold for a period of 20 min per day resulted in a significant acceleration in the healing process over a period of two weeks when compared with the control group. By day 14, the healing rate was ~2.74 mm^2^/day [112]. Furthermore, Low-Intensity Pulsed Ultrasound (LIPUS) is itself a clinical technology already approved by the Food and Drug Administration for promoting bone fracture healing [217]. When combined with piezoelectric dressings, LIPUS acts as a ‘wireless power source’ to excite the dressing to generate a local electric field, while simultaneously exerting inherent biological effects such as promoting cell proliferation and enhancing tissue permeability. This synergistic therapeutic mode demonstrates a clear and feasible pathway for clinical translation [218]. Figure 5c provides a visual representation of a piezo-photothermal composite dressing designed for utilization in infected wounds. In the US, the PLLA layer produces a piezoelectric effect (up to 1.78 V) that results in the release of ROS, which in turn kill bacteria. Furthermore, it has been demonstrated that Hsp90 expression is enhanced, thereby promoting cell proliferation and tissue repair, and accelerating the healing process of infected wounds. The piezoelectric group demonstrated a healing rate that was approximately three times faster than the control group over a period of 12 days. The healing process was found to be accelerated when both US and near-infrared (NIR) light were utilized concurrently.

#### Electrospinning of multifunctional synergistic piezoelectric materials

Electrospun piezoelectric nanofibers can also be integrated with functional materials, such as antimicrobial agents, growth factors and hydrogels, to construct trauma repair systems with active regulation capabilities [219, 220]. As demonstrated in Figure 5d, Chen *et al.* developed a bilayer catalytic membrane based on BTO/magnesium peroxide (MgO_2_) and PLGA nanofibers, with the outer layer coated with PDA. Upon excitation by US and NIR light, the membrane utilizes the piezoelectric and pyroelectric effects of BTO to generate polarization charges, which catalyze the decomposition of hydrogen peroxide to produce (-OH) radicals, thereby achieving rapid antibacterial action. Concurrently, the Mg^2+^ and oxygen released from the material have been shown to inhibit the nuclear factor kappa-B (NF-κB) inflammatory pathway, thereby promoting angiogenesis and collagen deposition, thus accelerating wound healing in a synergistic manner. The experimental results, both *in vitro* and *in vivo*, demonstrated that the membrane achieved a wound healing area of 94.04% by day 7 of treatment. This represents an increase of ~1.14-fold in comparison with the pure PLGA group, thus indicating a significant wound-healing promotion capability. In contrast, Wang *et al.* developed a hydroxyapatite/polylactic acid@polyvinylidene fluoride–hexafluoropropylene (HAP/PLA@PVDF-HFP) nanofiber-based wireless smart patch (Fig. 5e) [221]. The device utilizes the synergistic effect of piezoelectricity and triboelectricity to amplify the electrical signal output to 16.2 V, thereby combining the dual functions of wound healing and real-time monitoring. Following a 14-day treatment period, the wound healing rate was found to be 97.96%, thereby demonstrating its potential in the domain of smart medical dressings [222].

At present, piezoelectric nanofibers have not attained commercialization as standalone consumer commodities. Their practical application remains primarily confined to serving as functional components within specialized domains, including acoustic devices, sensors, and energy harvesting or conversion systems. Within the biomedical sphere, intelligent wound dressings, neural conduits, and bone repair scaffolds fabricated from piezoelectric nanofibers are currently the subject of active preclinical investigation. Preclinical animal studies have revealed notable therapeutic efficacy in tissue regeneration, thereby affirming the translational potential of this approach. The translation of this technology from bench to bedside faces three major challenges: the implementation of industrial-scale fabrication, the confirmation of long-term *in vivo* performance, and the development of corresponding regulatory frameworks for medical devices. With the progressive advancement of electrospinning technology and the continued momentum of interdisciplinary research. In the future, piezoelectric nanofiber-based medical products are projected to enter clinical trials, providing the clinical evidence necessary to substantiate their practical adoption.

### Future perspective

#### Clarifying the therapeutic mechanism of electrical stimulation

This review assesses the potential of local ES as a therapeutic application for enhanced wound healing. Research has demonstrated that ES therapy has a positive impact on every stage of skin wound healing. It fundamentally accelerates the tissue repair process through active biological modulation of the wound microenvironment, which includes regulating ROS levels, guiding macrophage polarization, enhancing antibacterial capacity, and mitigating inflammatory responses. However, its underlying mechanisms of action require further elucidation. Specifically: (i) an in-depth investigation of the therapeutic mechanisms of ES will help to clarify its key role in wound healing, thereby enabling a more precise matching of ES parameters with physiological responses. Current research often remains at the phenomenological level of therapeutic efficacy, making it difficult to achieve fundamental breakthroughs in pro-healing outcomes. (ii) Mechanistic research will help to reveal whether local ES promotes healing by modulating neural activity around the wound, thus leading to a deeper understanding of its biological essence. (iii) A deeper understanding of these mechanisms will also provide a theoretical basis and directional guidance for the material selection and structural design of current pro-healing dressings based on ES strategies.

#### Perfecting the systematic research framework for electrospun piezoelectric dressings

This review provides a systematic overview of the physical properties of various Electrospun Piezoelectric Materials, including their piezoelectric output, specific surface area, softness, and breathability. It specifically discusses the superior bioadaptability of their unique 1D nanofiber and 2D membrane structures to the complex wound microenvironment. These performance advantages, stemming from the intrinsic properties of the materials and their microstructure, constitute their core competitive edge over traditional dressings and pave the way for developing next-generation active therapeutic strategies for healing. The translation of these dressings from laboratory research to clinical application still presents several challenges. Notably, the evaluation of their wearing comfort lacks standardized and systematic criteria. Comfort, as a critical factor affecting patient compliance and therapeutic outcomes, pertains not only to the material's mechanical compatibility and breathability/moisture permeability but is also closely associated with interfacial behavior and skin response during prolonged wear. Therefore, establishing a scientific and effective evaluation system for comfort is an indispensable step in advancing electrospun piezoelectric dressings toward genuine clinical application.

#### Accelerating the clinical pathway translation of electrospun piezoelectric dressings

To advance the true clinical translation of electrospun piezoelectric dressings, this review systematically explores their biomedical applications in the field of wound healing. The inherent ES properties of this material enable the creation of a synergistic therapeutic platform for integration with drugs, magnetic components, or other piezoelectric materials. Whether by enhancing their intrinsic piezoelectric output or by constructing an electro-pharmacological synergistic therapeutic system, such composite strategies significantly surpass the pro-healing efficacy of monotherapies, demonstrating immense application potential. However, the in-depth development and optimization of all these combination strategies must be premised on a clear elucidation of their underlying therapeutic mechanisms. Future research must not only achieve breakthroughs at the mechanistic level but also actively benchmark against current clinical treatment standards and commercialized drug therapies to establish a comparative advantage in safety and efficacy. Only through this dual-pronged approach of ‘mechanism-guided’ and ‘clinically-benchmarked’ research can we fundamentally propel the next generation of electrospun dressings from the laboratory to the clinic, ultimately realizing their clinical application and industrialization.

## Conclusions

The critical role of skin as a protective barrier underscores the importance of advanced wound repair research. Electrospun piezoelectric nanofibers have emerged as a promising paradigm, harnessing micro-mechanical energy at the wound site to generate physiologically relevant electrical signals. This self-sustaining mechanism actively regulates the entire healing process. By synthesizing recent advances and addressing ongoing challenges, this review seeks to deepen the understanding of structure–function relationships in these materials, accelerate technological translation, and expand therapeutic options for complex wounds.

## Data Availability

The data used in this publication are available upon request.

## List of abbreviations

1D, one-dimensional; 2D, two-dimensional; 3D, three-dimensional; α-SMA, α-smooth muscle actin; AlN, aluminum nitride; AMPK, adenosine monophosphate-activated protein kinase; BTO, barium titanate; CXCR4, C-X-C chemokine receptor type 4; ECM, extracellular matrix; EGF, epidermal growth factor; EGFR, epidermal growth factor receptor; eNOS, endothelial nitric-oxide synthase; ERK, extracellular signal-regulated kinase; ES, electrical stimulation; FGF, fibroblast growth factor; FGF2, fibroblast growth factor2; HAP, hydroxyapatite; HFP, hexafluoropropylene; IL-1, interleukin-1; IL-1α, interleukin-1α; IL-6, interleukin-6; IL-8, interleukin-8; MAPK, mitogen-activated protein kinase; MgO_2_, magnesium peroxide; NF-κB, nuclear factor kappa-B; NIR, near-infrared; PCL, polycaprolactone; PDA, polydopamine; PDGFA, platelet-derived growth factors subunit A; PDGF, platelet-derived growth factor; PLA, polylactic acid; PLGA, poly (lactic-co-glycolic acid); PLLA, poly (l-lactic acid); PI3K, phosphatidylinositol 3-kinase; PU, polyurethane; PVA, polyvinyl alcohol; PVDF, polyvinylidene fluoride; PZT, piezoelectric ceramic transducer; ROS, reactive oxygen species; TGF-β, transforming growth factor-β; TGF-β1, transforming growth factor-β1; TiO_2_, titanium dioxide; TNF-α, tumor necrosis factor-α; SEM, scanning electron microscope; VEGF, vascular endothelial growth factor; ZnO, zinc oxide.
